# Effects of milk-derived bioactive peptide VPP on diarrhea of pre-weaning calves

**DOI:** 10.3389/fvets.2023.1154197

**Published:** 2023-03-29

**Authors:** Xiaomei Zong, Ya Gao, Yufeng Du, Jinxiu Hou, Linhai Yang, Qingbiao Xu

**Affiliations:** College of Animal Sciences and Technology, Huazhong Agricultural University, Wuhan, China

**Keywords:** VPP, gut inflammation, calf, diarrhea, gut microbiome

## Abstract

A well-known milk-derived bioactive tripeptide, VPP (Val-Pro-Pro) has good anti-inflammatory, anti-hypertension, and anti-hydrolysis properties. However, whether VPP can alleviate calf intestinal inflammation is unclear. In this experiment, the effects of VPP on growth, diarrhea incidence, serum biochemical indices, short-chain fatty acids, and fecal microorganisms were examined in pre-weaning Holstein calves. Eighteen calves with similar birth date, body weight, and genetic background were randomly assigned equally to two groups (*n* = 9). The control group was given 50 mL of phosphate buffer saline before morning feeding, whereas the VPP group received 50 mL of VPP solution (100 mg/kg body weight/d). The study lasted for 17 days, with the first 3 days used for adaptation. Initial and final body weights were determined, and daily dry matter intake and fecal score were recorded throughout the study. Serum hormone levels and antioxidant and immune indices were measured on day 14. Fecal microorganisms were collected on days 0, 7, and 14, and 16S rDNA sequencing was performed. Oral administration of VPP did not significantly affect calf average daily feed intake and body weight, but the growth rate in body weight was significantly higher in the VPP group than in the control group on day 7 (*P* < 0.05). Compared with the control, VPP significantly decreased serum TNF-α and IL-6 contents (*P* < 0.05), and concentrations of nitric oxide and IL-1β also decreased but not significantly (0.05 < *P* < 0.1). After seven days of VPP, relative abundances of *g_Lachnoclostridium, uncultured_bacterium_*, and *g_Streptococcus* in fecal samples increased significantly (*P* < 0.05). Compared with the control, VPP significantly increased concentrations of the fecal short-chain fatty acids n-butyric acid and isovaleric acid (*P* < 0.05). In conclusion, VPP can relieve intestinal inflammation and alleviate the degree of diarrhea in pre-weaning calves.

## 1. Introduction

As one of the most common diseases of young calves, diarrhea causes huge economic and productivity losses for cattle breeders (1). According to a survey released in 2018, diarrhea was responsible for 39% of calf deaths during the first 21 days of life (2). Antibiotics have long been used as a therapeutic agent for calf diarrhea, but antibiotic use in domestic animals is associated with many adverse effects, such as interference with host microorganisms, increased antibiotic resistance in hosts against antibiotic-resistant pathogens, and direct harm to the host (3). Therefore, suitable substitutes need to be identified.

A primary cause of diarrhea in newborn calves is enteropathic *Escherichia coli* infection. Enteropathic (*E*.) *coli* infection destroys proteins of intestinal compact junctions, releases cell-damaging toxins, damages small intestinal mucosa, and leads to mucosal inflammation, thus causing diarrhea in calves (4, 5). When pathogenic bacteria invade the intestinal tract of newborn calves, tight junction proteins are destroyed and inflammatory signaling pathways are activated, including those of mitogen-activated protein kinase, IkappaBalpha, and nuclear factor-kappaB. Pathogenic bacteria also induce production of interleukin-8 (IL-8), interleukin-6 (IL-6), and tumor necrosis factor-α (TNF-α) by small intestinal epithelial cells, which causes intestinal inflammatory response of calves (6). In addition, weaning stress may increase serum cortisol concentrations, which can be detrimental to cellular immunity and inhibit many non-specific immune responses, leading to increased disease susceptibility (7). Weaning stress increases contents of neutrophils, TNF-α, IL-6, and interleukin-1β (IL-1β) in blood of calves (8) and expression of apoptosis factors and pro-inflammatory factors including IL-1β, IL-8, interferon-γ, TNF-α, and toll like receptor 4, causing calf intestinal inflammatory response (9). However, a healthy intestinal environment of pre-weaning calves can alleviate weaning stress. Use of anti-inflammatory drugs can reduce the secretion of inflammatory factors and inhibit intestinal inflammatory response and thus alleviate the degree of diarrhea in calves (10).

The milk-derived bioactive peptide VPP (Val-Pro-Pro) has a positive anti-inflammatory effect in animal models of colitis (11). Dietary supplementation of VPP reduces expression of monocyte chemoattractant protein-1 and IL-6 and number of macrophages in inflammatory mice, as well as relieves adipose tissue inflammation through the mitogen-activated protein kinase-c-Jun N-terminal kinase pathway (12). The VPP peptide can also decrease expression of IL-1β and TNF-α, which alleviates inflammation in obese mice by activating angiotensin-converting enzyme (13). The tripeptide is highly resistant to gastric enzymes and pancreatic enzymes and rennet and is difficult to digest by digestive enzymes (14). Therefore, VPP that enters the digestive tract from the mouth can remain intact in the gut until it is absorbed. In addition, VPP can be transported in its intact form through the Caco-2 monolayer by paracellular diffusion, and the maximum concentration of VPP in animal blood can range from 10 to 100 μmol/L (15, 16). Therefore, VPP can be completely absorbed into the portal vein after ingestion and play a role in blood vessels. On the basis of previous research, it was hypothesized that VPP could relieve intestinal inflammation of calves and thus help to solve the problem of diarrhea. Therefore, an experiment was conducted with Holstein calves to determine the effects of VPP.

## 2. Materials and methods

### 2.1. Animals and management

The trial was conducted on the Linbo Cattle Farm, Jiangnan District, Nanning City, Guangxi Zhuang Autonomous Region, China, from June to July 2021. Eighteen 1-month-old Holstein calves with similar birth date, weight, and genetic background were randomly assigned equally to two groups (*n* = 9), with five male and four female calves in each group. Before morning feeding, 50 mL of phosphate buffer saline was given to the control (Ctrl) group and 50 mL of VPP solution (100 mg/kg body weight (BW)/d) was given to VPP group for 14 days. During the experiment, calves were housed in a single cage and fed 4 kg of milk substitute and 250 g of starter food every day, with free intake of food and water.

### 2.2. Data and sample collection

Electronic scales were used to record daily dry matter intake. Weights of calves were measured with a weighbridge on days 0, 7, and 14. At 2 h after morning feeding on days 10 and 14, 10 mL of blood was collected by the jugular vein. To isolate the serum, blood was centrifuged at 3,000 rpm for 10 min. The upper layer of serum was extracted with a pipetting gun and released into centrifuge tubes, which were stored at −20°C until analysis of serum biochemical indices. On days 0, 7, and 14, after stimulating the end of the rectum to cause defecation, 10 g of fresh feces was collected and packed into 5-mL sterile frozen tubes, which were then stored at −80°C.

### 2.3. Determination of diarrhea rate

Calves were observed daily for diarrhea, and feces scores were determined according to the four-point system. The fecal scoring criteria were according to Magalhaes et al. (17). When the feces score was ≥3, the diarrhea rate of calves was calculated according to the feces score.

### 2.4. Serum analyses

Bovine ELISA kits (NanJing JianCheng Bioengineering Institute, Nanjing, China) were used to determine serum biochemical indices, including activities of superoxide dismutase (SOD) and glutathione peroxidase (GSH-Px) and contents of malondialdehyde (MDA), immunoglobulin A (IgA), immunoglobulin G (IgG), IL-6, IL-8, IL-1β, endothelin 1 (ET-1), insulin-like growth factor 1 (IGF-1), TNF-α, and nitric oxide (NO).

### 2.5. Determination of fecal bacteria and short chain fatty acids

To analyze fecal bacterial composition and diversity, 16S rDNA amplicon sequencing was performed, with PCR amplification of bacterial 16S rDNA V3-V4 regions. After PCR products were evaluated and qualified, the library was constructed and sequenced by Beijing Baimaike Biotechnology Co., Ltd. (Beijing, China). Operational taxonomic unit (OUT) number, abundance, and Alpha diversity indices were obtained by analyzing fecal bacteria and principal coordinate analysis (PCoA) was conducted with OTU number. Referring to the detailed steps described in the literature (18), fecal short-chain fatty acids were analyzed by gas chromatography.

### 2.6. Statistical analyses

Data were analyzed by *t*-test, and figures were prepared using GraphPad Prism 9.0 (GraphPad Software, Inc., La Jolla, CA, USA). In all analyses, differences detected at *P* < 0.05 were considered significant.

## 3. Results

### 3.1. Effects of VPP on feed intake and body weight of pre-weaning calves

**Figure 1:**
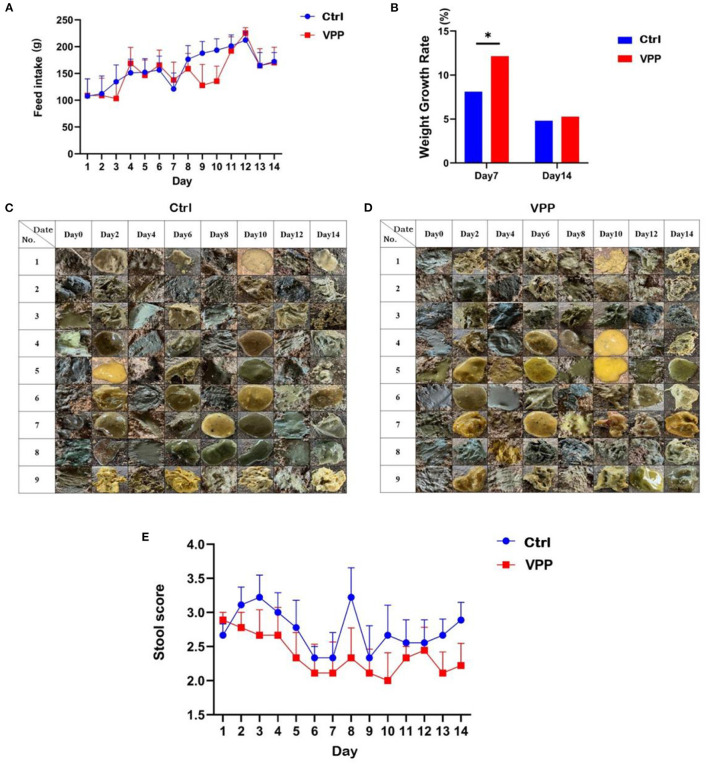
Effects of VPP on feed intake, body weight, and fecal score of pre-weaning calves. **(A)** Change in feed intake. **(B)** Growth rate in weight. **(C)** Control fecal sample. **(D)** VPP fecal sample. **(E)** Change in stool score. Values are means ± SD, *n* = 10. *Significant difference between group (*P* < 0.05).

Feed intake of calves in both Ctrl and VPP groups tended to increase (Figure 1A). Growth rate in body weight was significantly higher in the VPP group than in the Ctrl group only on d 7 (Figure 1B; *P* < 0.05).

### 3.2. Effects of VPP on fecal score of pre-weaning calves

The effect of VPP on the diarrhea rate of calves was not significant, and compared with the Ctrl group, the fecal score of calves in the VPP group after day 1 was lower throughout the study (Figures 1C–E).

### 3.3. Effects of VPP on serum biochemical indices of pre-weaning calves

**Figure 2:**
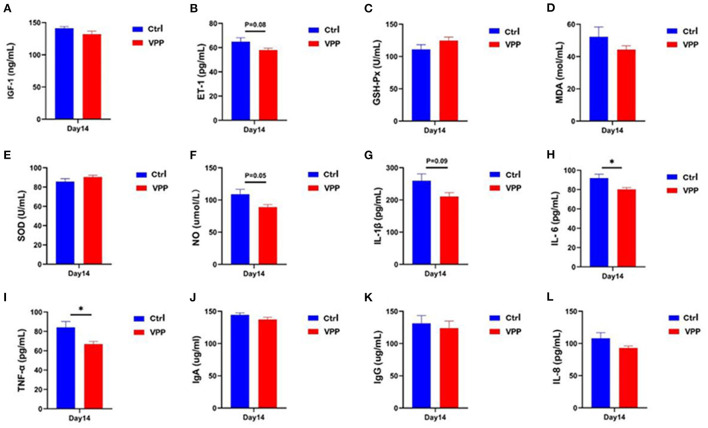
Effects of VPP on serum biochemical indices of pre-weaning calves. Concentrations of **(A)** insulin-like growth factor 1 (IGF-1), **(B)** endothelin 1 (ET-1), **(C)** glutathione peroxidase (GSH-Px), **(D)** malondialdehyde (MDA), **(E)** superoxide dismutase (SOD), **(F)** nitric oxide (NO), **(G)** interleukin-1β (IL-1β), **(H)** interleukin-6 (IL-6), **(I)** tumor necrosis factor-α (TNF-α), **(J)** immunoglobulin A (IgA), **(K)** immunoglobulin G (IgG), and **(L)** interleukin-8 (IL-8). Values are means ± SD, *n* = 10. *Significant difference between group (*P* < 0.05).

Serum concentration of IGF-1 in the VPP group was not significantly different compared with that in the Ctrl group (Figure 2A), but the concentration of ET-1 decreased, although not significantly (Figure 2B; 0.05 < *P* < 0.1). Differences in serum concentrations of GSH-P, MDA, and SOD between VPP and Ctrl groups were not significant (Figures 2C–E), although SOD and GSH-Px concentrations were higher in the VPP group. Serum NO concentration was not significantly lower in the VPP group than in the Ctrl group (Figure 2F; *P* = 0.05). Levels of IL-6 and TNF-α were significantly lower in the VPP group than in the Ctrl group (Figures 2H, I; *P* < 0.05), and level of IL-1β was also lower in VPP but the difference was not significant (Figure 2G, 0.05 < *P* < 0.1). Concentrations of IgA, IgG, and IL-8 were not significantly different between groups (Figures 2J–L).

### 3.4. Effects of VPP on short-chain fatty acids in pre-weaning calves

**Figure 3:**
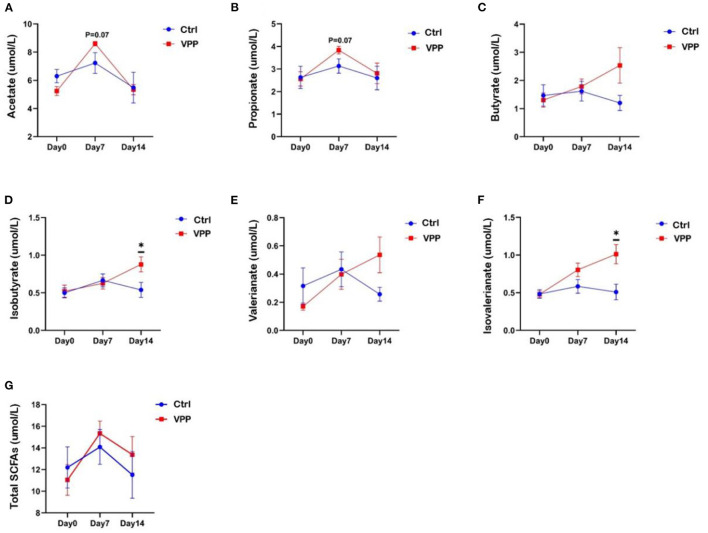
Effects of VPP on short-chain fatty acids in pre-weaning calves. Concentrations of **(A)** acetate, **(B)** propionate, **(C)** butyrate, **(D)** isobutyrate, **(E)** valerianate, **(F)** isovalerianate, and **(G)** total short-chain fatty acids (SCFAs). Values are means ± SEM, *n* = 10. *Significant difference between group (*P* < 0.05).

Fecal concentrations of butyric acid, isobutyric acid, valeric acid, and isovaleric acid in the VPP group increased throughout the study (Figures 3C–F). Concentrations of isobutyric acid and isovaleric acid were significantly higher in VPP group than in the Ctrl group on day 14 (Figures 3D, F; *P* < 0.05). On day 7, concentrations of acetic acid and propionic acid were higher in the VPP group than in the Ctrl group, but differences were not significant (Figures 3A, B; 0.05 < *P* < 0.1). The total short-chain fatty acid concentration was higher in VPP group than in the Ctrl group on days 7 and 14, but there was no significant difference (Figure 3G).

### 3.5. Effects of VPP on composition and diversity of fecal bacteria in pre-weaning calves

**Figure 4:**
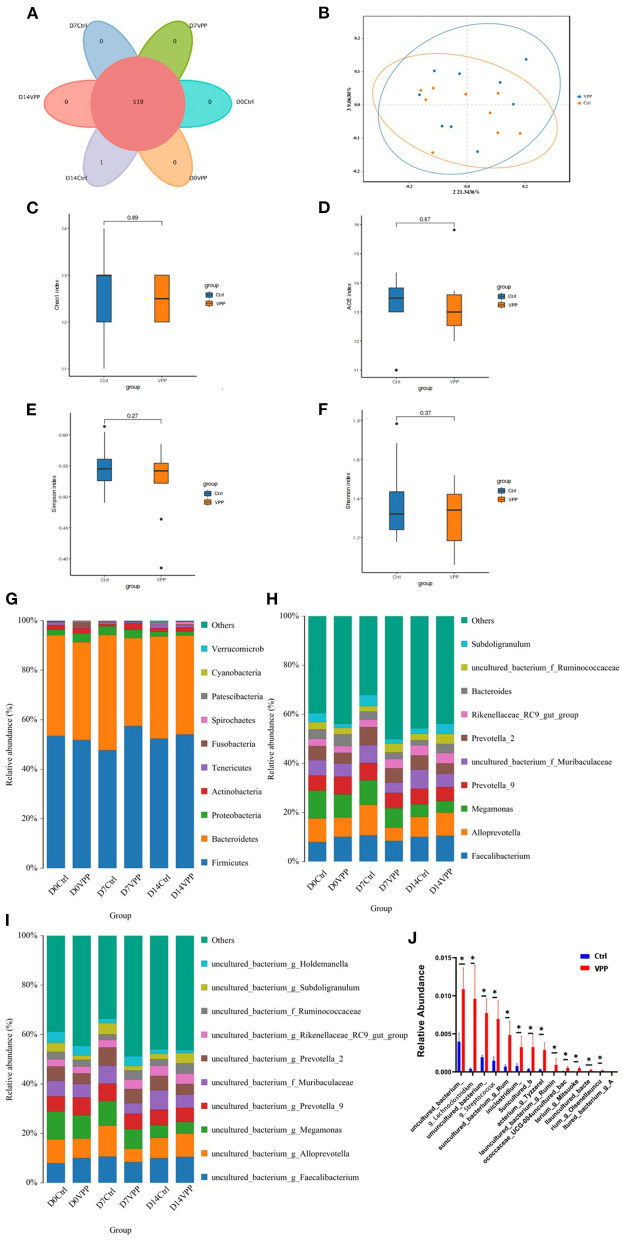
Effects of VPP on fecal bacteria of pre-weaning calves. **(A)** Venn analysis based on operational taxonomic units. **(B)** Principal coordinates analysis of fecal bacterial communities. Ctrl, control. Alpha diversity indices: **(C)** Chao1 index, **(D)** Ace index, **(E)** Simpson index, and **(F)** Shannon index. Composition of bacterial communities (relative abundance): **(G)** phyla, **(H)** genera, and **(I)** species. **(J)** Relative abundance of species of fecal bacteria.

Composition and diversity of fecal bacteria in each group were analyzed. In Ctrl and VPP groups, there were 519 common OTUs, and there was one unique OTU in the Ctrl group (Figure 4A). In addition, PCoA clearly indicated that composition of fecal bacterial communities was similar in Ctrl and VPP groups on day 14 (Figure 4B). Alpha diversity indices of fecal bacterial communities were compared between Ctrl and VPP groups on day 14, and differences in Chao1, ACE, Simpson, and Shannon indices were not significant, indicating similar species diversity between the groups (Figures 4C–F). Distributions of bacterial taxa at phylum, genus, and species levels were also analyzed. The dominant phyla of intestinal bacteria in Ctrl and VPP groups were *Firmicutes, Bacteroidetes, Proteobacteria, Actinobacteria*, and *Tenericutes*. In both groups on all days, the combined relative abundance of *Firmicutes* and *Bacteroidetes* was higher than 90%, and the proportion of *Firmicutes* in the VPP group exceeded 50% on the three sample dates (Figure 4G). The dominant genera of intestinal were *Faecalibacterium, Alloprevotella*, and *Megamonas* (Figure 4H), and the dominant species were *uncultured bacteria g Faecalibacterium, uncultured bacteria g Alloprevotella* and *uncultured bacteria g Megamonas* (Figure 4I). At different times, non-significant differences were observed between the two treatment groups among the 10 most abundant groups at phylum, genus, and species levels (Figures 4G–I). The 30 most abundant species of bacteria in samples collected on day 7 were analyzed, and relative abundances of *uncultured_bacterium, g_Lachnoclostridi, umuncul-tured_bacterium_* and *g_Streptococcu* were significantly different between Ctrl and VPP groups (Figure 4J; *P* < 0.05).

## 4. Discussion

Relatively low diarrhea rates and high feed intake and average daily gain in pre-weaning calves indicate good growth performance. In this experiment, oral administration of VPP significantly increased the weight gain rate of calves on day 7 and alleviated the degree of diarrhea. The mechanism for the decrease in diarrhea rate might be that VPP inhibited intestinal pathogenic bacteria and alleviated intestinal inflammation of lactating calves to some extent. In addition, VPP might have entered the blood by mediation of peptide transporter 1, which would improve serum immune indices, and alleviate the intestinal inflammatory response of calves, and thus alleviate the degree of diarrhea. Similarly, intravenous injection of the dipeptide Alanyl-glutamine in calves not only reduces the diarrhea rate caused by weaning but also improves feed intake and daily gain (19). Although not directly comparable with the results of this study, the different effects on feed intake and daily gain might result from differences in the type, function, and amino acid composition of the peptides between the two studies.

Complete forms of milk-derived active peptides can be absorbed and positively affect anti-oxidative, anti-hypertensive, antibacterial, anti-inflammatory, and immunomodulatory health-related properties (20). The pro-inflammatory factors IL-1β (21), IL-6 (22), and TNF-α (23) are involved in the inflammatory response, inducing changes in intestinal epithelial permeability. The serum results of this experiment are consistent with those associated with anti-inflammatory drugs used to relieve diarrhea of calves (10) and the concentrations of IL-6 and IL-8 were also significantly reduced in the study of using dimethylglycine to relieve intestinal damage caused by heat stress (24), indicating that VPP also decreases production of inflammatory factors and alleviates the inflammatory response. In this study, VPP decreased contents of the pro-inflammatory factors IL-1β, IL-6, and TNF-α in the blood of calves, demonstrating good anti-inflammatory effects.

In the PCoA in this study, there was little separation between VPP and Ctrl fecal bacterial communities on day 14, which indicated that VPP did not significantly affect the composition of fecal bacteria in calves. Recent studies have found that patients with inflammatory bowel disease all have an intestinal microbial imbalance. For example, alpha diversity decreases, and relative abundances of pathogenic bacteria, including *Gammaproteobacteria, Fusobacterium, Escherichia, Firmicutes*, and *Proteobacteria*, increase, whereas those of other bacteria, including *Bacteroides, Firmicutes, Clostridia, Ruminococcaceae, Bifidobacterium*, and *Lactobacillus*, decrease (25, 26). In this study, alpha diversity of fecal bacteria in the VPP group did not decrease significantly compared with that in the Ctrl group, and relative abundances of *Firmicutes* and *Proteobacteria* were not affected. In addition, no significant differences in bacteria at phylum, genus, and species levels were detected between groups in fecal samples collected on day 14. In samples collected on day 7, relative abundances of *uncultured_bacterium, g_Lachnoclostridium, uncultured_bacterium, g_Streptococcus*, and many other species were significantly different between VPP and Ctrl groups, but the differences were not maintained at the end of the experiment. Although some bioactive peptides can restrict harmful bacteria from colonizing intestinal epithelial cells (27) and promote intestinal homeostasis and counteract pathogens (28), in this trial, VPP did not show similar efficacy, which might be due to the strong hydrolytic resistance of VPP in the intestine.

As metabolites of intestinal microbes, short-chain fatty acids are important in interactions between intestinal microflora and the immune system (29). Short-chain fatty acids function in regulating tight junction protein expression, maintaining the activity of mucosal immune cells, lowering gut pH to suppress harmful bacteria, and protecting the intestinal epithelial barrier (30, 31). Short-chain fatty acids can suppress colon inflammation by activating butyric acid receptors and thereby inducing secretion of anti-inflammatory cytokines (32) and also by inhibiting inhibit the pro-inflammatory response of intestinal macrophages (33) and reducing the inflammatory response of peripheral blood mononuclear cells (34). On day 7, levels of propionic acid and acetic acid increased in the VPP group compared with those in the Ctrl group (0.05 < *P* < 0.1), but on day 14, contents were the same in the two groups. In addition, on day 14, isovaleric acid and isobutyric acid concentrations were significantly higher in the VPP group than in the Ctrl group (*P* < 0.05). Valeric acid can inhibit the growth of *Clostridium difficile* and thereby regulate intestinal homeostasis (35), whereas sodium butyrate can reduce IL-6 and TNF-α levels and thereby improve colitis in mice (36). Results of both studies are consistent with those of this test. Therefore, VPP might alleviate intestinal inflammation in diarrheal calves by altering gut flora to increase relative abundances of beneficial bacteria producing n-butyric acid and isovalerate.

## 5. Conclusions

The tripeptide VPP alleviated the degree of diarrhea in pre-weaning calves, by reducing contents of pro-inflammatory factors in the blood and thus relieving intestinal inflammation.

## Data availability statement

The original contributions presented in the study are included in the article/supplementary material, further inquiries can be directed to the corresponding author.

## Ethics statement

The animal study was reviewed and approved by Animal Welfare and Ethics Committee of Huazhong Agricultural University.

## Author contributions

XZ and YG prepared the original draft. YD, LY, and YG contributed to conception, supervised, data collection, conducted data analysis, and data curation. XZ performed investigation. YG designed methodology. XZ, YD, and QX reviewed and edited the manuscript. JH polished the article and improved the figures. All authors have read and agree to the published version of the manuscript.
